# The risk of gastrointestinal bleeding in patients taking third-generation P2Y12 inhibitors compared with clopidogrel: systematic review and meta-analysis

**DOI:** 10.1097/MS9.0000000000003821

**Published:** 2025-09-01

**Authors:** Sardar Muhammad Alamzaib, Muhammad Talha Maniya, Sara Hazaveh, Asad Ali Ahmed Cheema, Wania Sultan, Ahmad Qatanani, Marium Omair Mirza, Stacey Damito, Manoj Kumar, Syed Adeel Hassan, Tayyaba J Khan

**Affiliations:** a Department of Cardiology, Marshall University School of Medicine, Huntington, WV, United States; b Department of Medicine, Ziauddin University, Karachi, Pakistan; c Department of Medicine, Hackensack University Medical Center, Hackensack, NJ, United States; d International School of Medicine, International University of Kyrgyzstan, Bishkek, Kyrgyzstan; e Department of Medicine, Dow University of Health Sciences, Karachi, Pakistan; f Department of Medicine, John H. Stroger Jr. Hospital of Cook County, Chicago, IL, United States; g Department of Digestive Diseases and Nutrition, University of Kentucky, Lexington, KY, United States; h Department of Medicine, Liaquat University of Medical and Health sciences, Sindh, Pakistan

**Keywords:** anti-platelet therapy, clopidogrel, gastrointestinal bleeding, third-generation P2Y12 inhibitors

## Abstract

**Background:**

Dual antiplatelet therapy is an essential component in the management of acute coronary syndrome (ACS) but with a significant risk of bleeding. Newer third-generation P2Y12 inhibitors have proven to be more efficacious but have increased the risk of bleeding. This meta-analysis will study the difference in risk of gastrointestinal bleeding in clopidogrel vs. third-generation oral P2Y12 inhibitors.

**Methods:**

A literature search was done in two databases (PubMed/Medline and Cochrane Central). All studies meeting the inclusion criteria assessing the occurrence of gastrointestinal bleeding following the use of oral third-generation P2Y12 inhibitors or clopidogrel were systematically identified. A random-effects meta-analysis evaluated the two arms’ risk ratios (RR).

**Results:**

A total of 16 studies were included in our analysis, 11 of which compared ticagrelor to clopidogrel and 5 of which compared prasugrel to clopidogrel. The combined risk for both third-generation P2Y12 inhibitors, Ticagrelor and Prasugrel, was as follows [RR: 1.31 (1.15–1.49); *P* < 0.0001]. Heterogeneity was reported to be *I*^2^ = 4%.

**Conclusion:**

Oral third-generation P2Y12 inhibitors were associated with an increased occurrence of gastrointestinal bleeding compared to clopidogrel.

## Introduction

Acute coronary syndrome (ACS) is a condition associated with substantial morbidity and mortality, accounting for a significant proportion of cardiovascular disease-related deaths^[1]^. The current preferred treatment standard is dual antiplatelet therapy (DAPT), i.e., aspirin with a P2Y12 inhibitor (ticagrelor, prasugrel, or clopidogrel)^[2,3]^. Together, the combined action of aspirin and a P2Y12 inhibitor in DAPT targets different pathways involved in platelet activation, effectively reducing platelet aggregation. In doing so, this regimen aims to prevent significant adverse cardiovascular or cerebrovascular events (MACE).

**HIGHLIGHTS**

- Ticagrelor, when compared with clopidogrel in our pooled analysis, showed an increased risk of gastrointestinal bleeding.
- The use of the third-generation P2Y12 inhibitor Prasugrel in comparison with clopidogrel was also associated with an increased risk of gastrointestinal bleeding.
- Considering the potential benefits of clopidogrel in reducing the risk of GI bleeding, it may be a preferable option for patients with specific gastrointestinal conditions such as inflammatory bowel syndrome.

Ticagrelor and prasugrel, classified as third-generation P2Y12 inhibitors, exhibit several advantages over clopidogrel. They demonstrate quicker onset of action and reversible (ticagrelor only) effects, enabling prompt reversal if necessary^[4]^. Additionally, ticagrelor and prasugrel achieve a more potent and consistent antiplatelet effect at lower dosages than clopidogrel^[5]^.

However, the increased efficacy in preventing clot formation of prasugrel and ticagrelor comes with a trade-off of an increased risk of bleeding events^[6,7]^. The Platelet Inhibition and Patient Outcomes Trial (PLATO) was a large-scale clinical trial conducted to compare the effectiveness and safety of ticagrelor versus clopidogrel in patients with ACS^[8]^. It is important to note that they found a higher incidence of non-coronary artery bypass grafting (non-CABG) major bleeding events in patients treated with ticagrelor than clopidogrel. The most common site of bleeding reported in the trial was the gastrointestinal tract. Unfortunately, there is a lack of comprehensive studies specifically focusing on the statistical implications of gastrointestinal bleeding associated with the use of oral third-generation P2Y12 inhibitors.

A previous meta-analysis^[9]^, evaluated the risk of gastrointestinal bleeding associated with third-generation P2Y12 inhibitors compared to clopidogrel. While that analysis included more studies overall, only a subset reported gastrointestinal bleeding as a clearly defined outcome, and the study incorporated data from post-hoc analyses and clinical trial registries, which may have lacked methodological uniformity. In contrast, the present review includes fewer studies (*n* = 16) but features a larger total patient population (67 000 vs. 58 678) and is limited exclusively to randomized controlled trials (RCTs) with predefined, adjudicated gastrointestinal bleeding outcomes. This methodological rigor enhances the internal validity, consistency, and clinical interpretability of our findings compared to prior reviews. In addition, recent observational studies have presented contrasting findings to those drawn in the previous meta-analysis^[10,11]^. Consequently, there is a need to update and expand upon this topic in light of more recent evidence^[12–15]^.

This meta-analysis aims to provide the most up-to-date information in the form of randomized control trials with substantial sample sizes, which increase the statistical power of the analysis, allowing for more accurate estimation of treatment effects and minimizing the impact of random variation. Longer follow-up times allow for the evaluation of both short-term and long-term effects, providing a more thorough understanding of the benefits and risks associated with the interventions over an extended period. This is particularly important in assessing outcomes that may manifest or evolve. This updated analysis, thereby, aims to strengthen the reliability and accuracy of the findings by incorporating evidence within the framework of the current literature.

## Methods

### Literature search strategy and data sources

We systematically searched for published RCTs of ticagrelor or prasugrel versus clopidogrel on PubMed and Cochrane Library until May 2023. The primary outcome was the risk of GI bleeding among users of oral third-generation P2Y12 inhibitors (i.e., prasugrel and ticagrelor) when compared to clopidogrel, expressed as risk ratio (RR) and 95% confidence interval (CI). Mesh terms and Boolean operators were used to devise an effective search strategy for each database (Supplemental Digital Content Table 1, available at: http://links.lww.com/MS9/A923). Since this study utilizes publicly available data, study registry and IRB approval were also unnecessary. This review was prospectively registered on PROSPERO (CRD420241105537).

### Study selection

After a systematic search, the articles identified were exported to the EndNote Reference Library, where duplicates were removed from the dataset. The remaining publications were subjected to initial screening based on their title and abstract as per PRISMA guidelines^[16]^. Subsequently, the full text of the articles was thoroughly reviewed to determine their relevance. Two reviewers (S.M.A. and S.D.) conducted the screening process independently. A third researcher (T.S.) was consulted in the case of any disagreement. Articles were included for further analysis if they satisfied all of the predetermined inclusion and exclusion criteria.

### Inclusion/exclusion criteria

Studies were included if they were: (i) RCTs; (ii) compared third-generation P2Y12 inhibitors, prasugrel, and/or ticagrelor with clopidogrel; (iii) had GI bleeding as one of their outcomes. (iv) Only full-text articles published in English were included in the analysis. Studies were excluded if: (i) the initial data provided lacked comprehensive information, such as the number of patients or events in each intervention group. (ii) The definition and severity of the bleeding events were not clearly specified. (iii) The delivery of study drugs was combined with the use of anticoagulants. (iv) The inclusion criteria involved nonhuman subjects or restricted the study to healthy individuals. (v) Focused exclusively on who suffered from either chronic renal disease or any bleeding disorder.

### Data extraction and quality assessment

The primary focus of this study was to examine the occurrence of GI bleeding following the use of oral third-generation P2Y12 inhibitors or clopidogrel. Various aspects were considered during this analysis, including study characteristics, publication date, study location, sample size, dosage, and duration of follow-up. The baseline characteristics of the patients enrolled in the studies were also considered. Outcome data, and safety data, were extracted for analysis. The quality assessment of the included RCTs was conducted using the Cochrane Risk of Bias tool^[17]^.

### Statistical analysis

The statistical analysis was conducted using Review Manager (Version 5.3 Copenhagen: The Nordic Cochrane Centre, The Cochrane Collaboration, 2014) for all analyses. The trial results were presented using RR with corresponding 95% CIs, and a random effects model was utilized to combine the data. Forest plots were created to visually assess the mixed results, allowing for an easy comparison of the outcomes across different studies. Additionally, the chi-square test was applied to evaluate significant differences among subgroups. Statistical significance was defined as a *P*-value of less than 0.05 in all instances. This study assessed heterogeneity among the included studies using Higgins *I*^2^ values. *I*^2^ values of less than 50% were categorized as indicating low heterogeneity, values between 50% and 75% showed moderate heterogeneity, and values exceeding 75% were regarded as high heterogeneity. Begg’s test was conducted to evaluate publication bias, and the funnel plot was visually inspected.

## Results

### Search

Following our search, we identified 257 potential studies. Through carefully evaluating of these studies based on our inclusion and exclusion criteria, we narrowed down the selection to 16 RCTs for analysis. The PRISMA flow chart (Supplemental Digital Content Table 1, available at: http://links.lww.com/MS9/A923) summarizes the search and study selection process. The meta-analysis included a total study population of 67 000 patients undergoing antiplatelet therapy, with 33 169 patients in the clopidogrel arm, 12 407 patients in the prasugrel arm, and 21 431 patients in the ticagrelor arm. The baseline characteristics of the included studies are presented in Tables 1 and 2. The symmetrical funnel plot indicates the absence of publication bias. The results of our meta-analysis are presented in a detailed forest plot. Our comprehensive search found that GI bleed occurred in 606 patients (1.8%) treated with third-generation P2Y12 inhibitors and 454 patients (1.36%) treated with clopidogrel. We also conducted a sensitivity analysis.

**Table 1:** Baseline characteristics of studies involving ticagrelor

Study, year	Country or area	Indications	Population of ticagrelor studies	Population of clopidogrel studies	Range of age	Intervention (mg, once)/MD (mg, QD)	Control (mg, once)/MD (mg, QD)	Exposure duration (Median)	Follow-up duration (Median)
Husted, 2006	Denmark, Hungary and Norway	CAD or artery diseases	164	37	25-85	Ticagrelor, LD —/50-400	Clopidogrel LD —/75	28 d	28 d
DISPERSE-2, 2007	14 countries	NSTE-ACS	657	327	≥18	Ticagrelor, LD —/90-180	Clopidogrel LD 300/75	4–12 wk (56 d)	12 wk
PLATO, 2009	43 countries	ACS	9235	9186	≥18	Ticagrelor, LD 180/90	Clopidogrel LD 300-600/75	12 mo (277 d)	12 mo
Hiasa, 2014	Japan, Philippine	PCI or ACS	93	46	20-80	Ticagrelor, LD —/45-90	Clopidogrel LD —/75	28 d	8 wk
Tang, 2016	China	STEMI undergoing PCI	210	210	>18	Ticagrelor, LD 180/90	Clopidogrel LD 600/75	12 mo	6 mo
Yao, 2017	China	AMI undergoing PCI	60	60	47-72	Ticagrelor, LD 180/90	Clopidogrel LD 600/75	—	6 mo
EUCLID, 2017	28 countries	Peripheral artery disease	6910	6932	≥50	Ticagrelor, LD —/90	Clopidogrel LD —/75	—	30 d
Li, 2018	China	STEMI and PCI	161	281	≥18	Ticagrelor, LD 180/90	Clopidogrel LD 600/75	_	12 mo
Wang, 2019	China	Minor ischemic stroke/Transient ischemic attack	336	339	54.0–67.0	Ticagrelor, LD 180/90	Clopidogrel LD 300/75	90 days	6 mo
Park, 2019	Korea	ACS with or without ST elevation	400	400	≥18	Ticagrelor, LD 180/90	Clopidogrel LD 600/75	12 mo	1 mo
Wang, 2021	China	Ischemic stroke/ High risk Transient ischemic attack	3205	3207	56.9–71.7	Ticagrelor, LD 180/90	Clopidogrel LD 300/75	3 mo	9 mo

ACS – acute coronary syndrome, AMI – acute myocardial infarction, CAD – coronary artery disease, d – days, LD – loading dose, MD – maintenance dose, mo – months, NSTE-ACS - non-ST-elevation acute coronary syndrome, PCI – percutaneous coronary intervention, QD – once daily, STEMI – ST-Elevation Myocardial Infarction, wk – weeks

**Table 2:** Baseline Characteristics of Studies Involving Prasugrel

Study, year	Country or area	Indications	Population of Prasugrel studies	Population of Clopidogrel studies	Range of age	Intervention (mg, once)/MD (mg, QD)	Control (mg, once)/MD (mg, QD)	Exposure duration (Median)	Follow-up duration (Median)
TRITON-TIMI, 2007	30 countries	ACS	6741	6716	≥18	Prasugrel, LD 60/10	Clopidogrel LD 300/75	6-15 (14.5) mo	6-15 mo
Ge, 2010	4 countries or areas	ACS	463	229	≥18	Prasugrel, LD 30-60/5-10	Clopidogrel LD 300/75	90 d	90 d
TRILOGY-ACS, 2012	52 countries	Unstable angina or NSTEMI without revascularization	210	210	≥18	Prasugrel, LD 30/5-10	Clopidogrel LD 300/75	6-30 (14.8) mo	6-30 (17.1) mo
TRIGGER-PCI, 2012	Germany and USA	Stable CAD undergoing PCI with HTPR	4623	4617	18-80	Prasugrel, LD 60/10	Clopidogrel LD 600/75	—	3 or 6 mo
PRASFIT-Elective, 2014	Japan	PCI with HTPR	370	372	≥20	Prasugrel, LD 20/3.75	Clopidogrel LD 300/75	24-48 wk	14 d after the last dose

ACS—Acute Coronary Syndrome, CAD—Coronary Artery Disease, d—Days, HTPR—High on-Treatment Platelet Reactivity, LD—Loading Dose, MD—Maintenance Dose, mo—Months, NSTEMI—Non-ST-Elevation Myocardial Infarction, PCI—Percutaneous Coronary Intervention, QD—Once Daily, wk—Weeks.

### Results of meta-analysis

**Figure 1.**
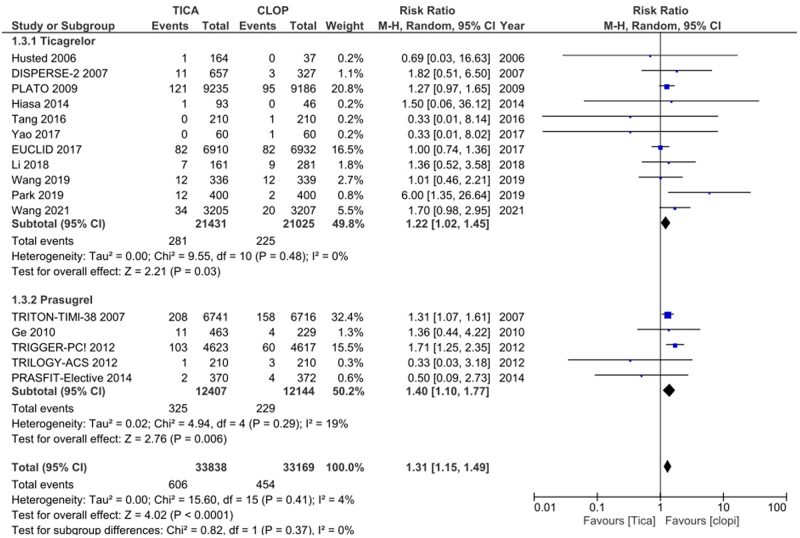
Forest plot of gastrointestinal bleeding events with any oral third generation P2Y12inhibitor compared to clopidogrel.

**Figure 2.**
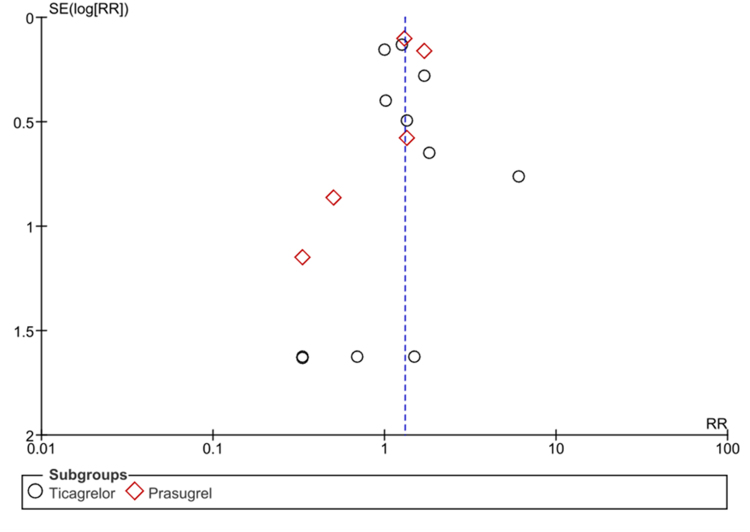
Funnel plot of included studies.

**Figure 3.**
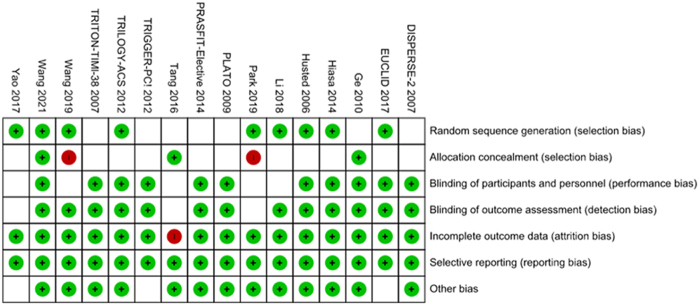
Risk of bias summary.

**Figure 4.**
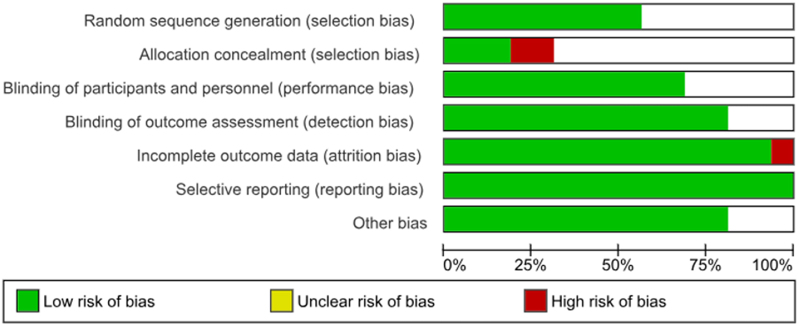
Risk of bias graph.

After analysis of the 16 RCTs that met our inclusion criteria, the outcome of gastrointestinal bleeds related to Ticagrelor and Prasugrel was as follows [RR: 1.31 (1.15–1.49); *P* < 0.0001]. Heterogeneity was reported to be *I*^2^ = 4%. The results are summarized in Figure 1. A funnel plot can be seen in Figure 2, and the risk of bias summary and graph in Figures 3 and 4, respectively.

#### Ticagrelor

**Figure 5.**
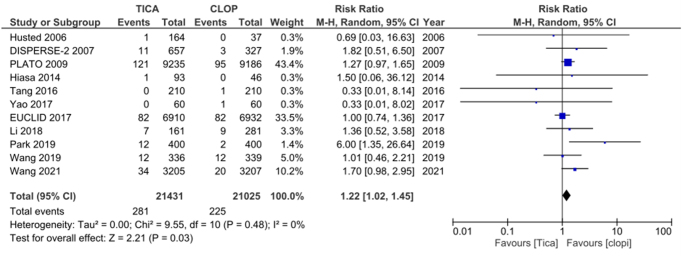
Forest plot of gastrointestinal bleeding events with ticagrelor compared to clopidogrel.

Among the 16 RCTs analyzed, 11 reported GI bleeding outcomes for ticagrelor. Consistent with the previous findings, Ticagrelor, when compared with clopidogrel in our pooled analysis, showed an increased risk of gastrointestinal bleeding [RR: 1.22 (1.02–1.45); *P* = 0.03]. Heterogeneity was reported to be *I*^2^ = 0%. The results are summarized in Figure 5.

#### Prasugrel

**Figure 6.**
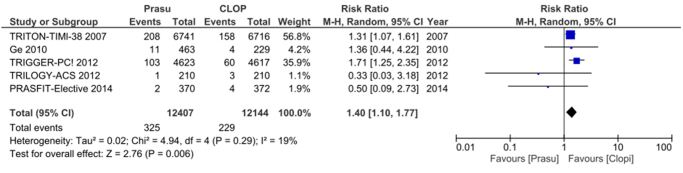
Forest plot of gastrointestinal bleeding events with prasugrel compared to clopidogrel.

Among the 16 RCTs analyzed, 5 reported GI bleeding outcomes for Prasugrel. Our results demonstrated that the use of the third-generation P2Y12 inhibitor prasugrel in comparison with clopidogrel was also associated with an increased risk of gastrointestinal bleeding [RR: 1.40 (1.10–1.77); *P* = 0.006], heterogeneity was reported to be *I*^2^ = 19%. The risk appeared to be even more pronounced than in ticagrelor. The results are summarized in Figure 6.

## Discussion

In this pooled analysis, comprising a total of 16 studies involving a substantial population of approximately 60 000 patients, our objective was to enhance the current body of literature by incorporating recent trials of significant sample sizes and extended follow-up durations^[12–15]^. Given that GI bleeding has been identified as the prevailing form of bleeding after antiplatelet therapy^[8]^, our comprehensive analysis aimed to investigate the relationship between the utilization of third-generation P2Y12 inhibitors and the frequency of GI bleeds compared to clopidogrel. Our meticulous analysis confirms that the administration of ticagrelor or prasugrel exhibited a heightened incidence of GI bleeds. These findings align consistently with the conclusions from preceding meta-analyses on this topic^[9]^.

Our analysis yielded more statistically significant results than to the previous meta-analysis, demonstrating a stronger association between ticagrelor use and an increased risk of GI bleeding. These findings provide more robust evidence supporting the higher likelihood of GI bleeding with ticagrelor than the previous analysis. It aligns with current literature and emphasizes the importance of considering this elevated risk when prescribing ticagrelor to patients. Another noteworthy aspect of our findings is the low heterogeneity observed across all analyses. This low heterogeneity is a positive outcome as it signifies a high agreement and consistency among the included studies. The reduced heterogeneity strengthens the reliability and robustness of our findings, lending greater credibility to the observed association between third-generation oral P2Y12 inhibitor use and the risk of gastrointestinal bleeding.

Numerous recent studies have reinforced this particular finding; one study conducted by Wang Y et al^[12]^, supports this conclusion further despite the absence of statistically significant differences in the risk of severe or moderate bleeding between the two treatment groups (ticagrelor and clopidogrel). It was observed that ticagrelor demonstrated a higher occurrence of GI bleeds (1.6%) than clopidogrel (0.6%)^[12]^. These findings shed light on the potential variations in bleeding risks associated with these medications and emphasize the importance of considering the specific risks and benefits when selecting an antiplatelet therapy.

Additionally, in a cohort study conducted in 2020^[17]^, the researchers analyzed 3970 reports from the Japanese Adverse Drug Event Report (JADER) database, spanning the years 2014 to 2017. Among these reports, 518 were associated with prasugrel, while 3452 were linked to clopidogrel. They also examined 91 914 reports from the FDA Adverse Event Reporting System (FAERS) between 2009 and 2019. Of these reports, 5992 were related to prasugrel, and 85 922 were related to clopidogrel. The study concluded that prasugrel exhibited a higher risk of GI bleeds compared to clopidogrel, with a RR of 2.27 (95% CI: 1.73–2.97) in JADER and 1.41 (95% CI: 1.29–1.54) in FAERS^[17]^. Another observational study done by Wang et al^[18]^, found that among patients with high bleeding risk (HBR) who experienced ACS, the administration of ticagrelor, when compared to clopidogrel, was associated with an increased incidence of GI bleeding during their hospital stay [OR: 1.61 (1.16, 2.25) *P* = 0.004]. Nonetheless, it is worth noting that the use of ticagrelor did not significantly reduce major adverse cardiovascular and cerebrovascular events (MACCE) within the same timeframe. These findings highlight the importance of carefully considering the risk-benefit profile when selecting antiplatelet therapies for ACS patients with high bleeding risk^[18]^.

Consistent with the previous meta-analysis, our analysis demonstrated a heightened risk of Prasugrel-related GI bleeds. This result further confirms the increased likelihood of GI bleeding in patients who were treated with Prasugrel when compared to other treatment options.

However, inconsistent with the present findings, some observational studies show that there is no statistical difference in GI bleeding between third-generation P2Y12 inhibitors compared to clopidogrel. As seen in a study analyzing a Japanese database, the bleeding between the use of prasugrel and clopidogrel was not seen to be statistically significant^[10]^. In one study^[11]^, involving a total of 37 019 patients, the comparison between clopidogrel and ticagrelor revealed a 34% reduction in GI bleeds, with a hazard ratio (HR) of 0.66 (95% CI: 0.54–0.81), observed for GI bleeding overall and in patients with non-ST-segment elevation acute coronary syndrome (NSTE-ACS). Additionally, ticagrelor demonstrated a 37% reduction in GI bleeding risk (HR 0.63; 95% CI: 0.42–0.93) specifically in patients with ST-segment elevation myocardial infarction (STEMI). On the other hand, when compared to clopidogrel, prasugrel was associated with an overall 21% reduction in GI bleeding risk (HR 0.79; 95% CI: 0.64–0.97). In STEMI patients, prasugrel exhibited a 36% reduction in GI bleeding risk (HR 0.64; 95% CI: 0.49–0.85). However, no significant reduction in GI bleeding risk was observed for prasugrel use in patients with NSTE-ACS^[11]^.

However, these findings were derived from an observational cohort study based on administrative claims data and diagnostic coding, rather than from randomized controlled trial data. This study design introduces the possibility of misclassification bias and residual confounding, particularly confounding by indication where patients perceived to be at higher bleeding risk may have been more likely to receive clopidogrel. Additional confounding factors in the Abraham study include the inclusion of a heterogeneous patient population across a broad age range, varied comorbidities, and concurrent use of medications known to affect bleeding risk (e.g., NSAIDs, corticosteroids, anticoagulants). These variables were not controlled for through randomization and may have independently contributed to gastrointestinal bleeding outcomes. In contrast, our meta-analysis includes only RCTs with prospectively defined and adjudicated GI bleeding outcomes, thereby minimizing such biases. Furthermore, our sample size is larger and more balanced across treatment groups (ticagrelor: 19 053; prasugrel: 14 006; clopidogrel: 33 948), compared to Abraham et al (ticagrelor: 7340; prasugrel: 5847; clopidogrel: 26 381). These differences in study design, outcome classification, and population characteristics likely explain the observed discrepancy between our findings and those of Abraham et al (2020)^[11]^.

It is quite plausible that by effectively inhibiting platelet function, these medications reduce the clotting ability of blood, which can disrupt the standard clotting mechanisms in the GI tract and heighten the likelihood of bleeding. Additionally, ticagrelor and prasugrel have been associated with increased gastric acid secretion in some individuals. Elevated levels of gastric acid can irritate and erode the lining of the GI tract, making it more vulnerable to bleeding. Furthermore, the long-term use of antiplatelet medications like ticagrelor and prasugrel can raise the risk of gastric and duodenal ulcer formation. These ulcers can compromise the protective lining of the GI tract, further increasing the susceptibility to bleeding. While GI bleeding is a relatively rare side effect, it is important to monitor for symptoms and consult with healthcare professionals to address any concerns and manage the associated risks effectively.

This study is subject to certain limitations, one notable observation is that many of the recent RCTs did not provide statistical reporting specifically for GI bleeds. During our literature search, we encountered gaps in the available research regarding specific bleeding locations associated with third-generation P2Y12 inhibitors and clopidogrel use. Many studies often categorized bleeding events as minor or major without providing detailed information about the particular locations of the bleeds. This lack of granularity limited our ability to perform a more comprehensive analysis of the bleeding events and understand the specific nature of the bleeding associated with these medications. It is crucial to highlight that there was a lack of uniformity in the dosages of P2Y12 inhibitors employed across the studies. Accurate assessment of bleeding outcomes requires careful consideration of the dosage as a crucial factor. It is imperative to standardize the dosage to obtain precise insights into the occurrence and severity of bleeding events. Establishing consistent dosage protocols across studies would enable more meaningful comparisons and enhance our understanding of the relationship between medication dosing and bleeding risk. This standardized approach would contribute to better-informed decision-making regarding the use of antiplatelet medications and the management of potential bleeding complications.

To conclude, this meta-analysis illustrates that the utilization of oral third-generation P2Y12 inhibitors results in an increased occurrence of gastrointestinal bleeding compared to their older counterpart, clopidogrel. Since there was a paucity of data in statistically quantifying GI bleeds, further investigations are required to establish a comprehensive body of evidence regarding the comparative impact of clopidogrel and newer generation P2Y12 inhibitors (Ticagrelor and Prasugrel). These studies should encompass larger sample sizes, and more randomized controlled trials should include this data in their supplementary material and extend the duration of follow-up to ensure the reliability and validity of findings. Considering the potential benefits of clopidogrel in reducing the risk of GI bleeding, it may be a preferable option for patients with specific gastrointestinal conditions such as IBS (irritable bowel syndrome). By incorporating these findings into clinical practice, care providers can optimize patient care and improve outcomes.

## Supplementary Material

## Data Availability

All data generated or analyzed during this study are publicly available.

## Research registration unique identifying number (UIN)

This systematic review was registered on PROSPERO (CRD420241105537): https://www.crd.york.ac.uk/prospero/display_record.php?ID=CRD420241105537.
