# Supplementary material for: The risk of gastrointestinal bleeding in patients taking third-generation P2Y12 inhibitors compared with clopidogrel: systematic review and meta-analysis
Source: Ann Med Surg (Lond). 2025 Sep 1;87(10):6694–701. doi: 10.1097/MS9.0000000000003821 (PMC12577880; doi:10.1097/MS9.0000000000003821)
Supplement: Supplementary file 1 [file ms9-87-6694-s001.docx]

**Supplemental Table 1:** Search strategy used in each database searched

| **Database (Articles Retrieved)** | **Search Strategy** |  |
| --- | --- | --- |
| PUBMED (257 results) | (Prasugrel OR “Cs-747” OR Ly640315 OR Ticagrelor OR Azd6140 OR “Purinergic P2Y Receptor Antagonists”[Mesh] OR “Prasugrel Hydrochloride”[Mesh] OR “Ticagrelor”[Mesh] OR “P2y12 inhibitor” OR “P2y12 inhibitors”) AND (clopidogrel OR “Clopidogrel”[Mesh]) AND (“Gastrointestinal Hemorrhage”[Mesh] OR “Hemorrhage”[Mesh] OR bleed* OR hemorrhage* OR hemorrhage* OR blood) |  |
|  |  |  |
|  |  |  |
|  |  |  |
|  |  |  |
|  |  |  |
| Cochrane Central (200 results) | (Prasugrel OR “Cs-747” OR Ly640315 OR Ticagrelor OR Azd6140 OR “Purinergic P2Y Receptor Antagonists”[Mesh] OR “Prasugrel Hydrochloride”[Mesh] OR “Ticagrelor”[Mesh] OR “P2y12 inhibitor” OR “P2y12 inhibitors”) AND (clopidogrel OR “Clopidogrel”[Mesh]) AND (“Gastrointestinal Hemorrhage”[Mesh] OR “Hemorrhage”[Mesh] OR bleed* OR hemorrhage* OR hemorrhage* OR blood) |  |
|  |  |  |
|  |  |  |
|  |  |  |
|  |  |  |
